# A Study of Liposome Structure Changes with Temperature Using Non-Equilibrium Molecular Dynamics Simulations

**DOI:** 10.3390/membranes16040124

**Published:** 2026-03-31

**Authors:** Gary Q. Yang, Weibin Cai, Ying Wan

**Affiliations:** 1 College of Bioscience and Bioengineering, Jiangxi Agricultural University, Nanchang 330045, China; 2 School of Chemical and Environmental Engineering, China University of Mining and Technology, Beijing 100083, China

**Keywords:** liposome, molecular dynamics simulations, liposome structure change with temperature, MARTINI force field

## Abstract

Liposomes, spherical bilayer lipid-containing vesicles, are promising nanocarriers used for constructing drug delivery systems (DDS). Various strategies can be employed to loosen or break the liposome and release drugs as the tumor cells-targeting DDS made of liposomes reach the targeted sites. One of the most commonly used strategies is to heat the liposomal DDS by letting the gold nanoparticles or other light-absorbing substances that partition in various portions (inner water core, lipid bilayer or outside) of the liposome absorb light irradiation. Then, which portion can lead to the largest liposome structure change due to the same temperature variation? The answer is essential to aid the design of liposomal DDS; thus, wet lab experiments were carried out. However, even though irradiation-absorbing substances in different portions were irradiated for the same time and with the same irradiation intensity, it was impossible to ensure the three portions have the same temperature increase in the experiments. Furthermore, it is impossible to learn the related micromechanism and molecular-level details of the effects of temperature changes on the liposome structure with experimental methods. The molecular dynamics (MD) method is extensively employed by researchers to obtain in-depth molecular-level insights. Most researchers tend to simulate only a planar lipid bilayer structure, but Amărandi et al. demonstrated that such simplification strategy may give wrong simulation results contrary to the experimental results. Though Jämbeck et al. and Zhu et al. established whole spherical liposome systems with a diameter of about a dozen nanometers and simulated the systems with MD simulations, they did not simulate temperature-relevant properties of the liposome. Therefore, currently there is a lack of research on simulating the structure change in a whole spherical liposome due to temperature variations. So, we established the whole spherical structure of the liposome, simulated how it changes with temperatures and obtained molecular-level research results. It is observed that the temperature increase in the lipid bilayer causes the largest increase in lipid strand sway amplitude, the largest changes in lipid positions, the largest decrease in the distribution density of lipids and water around a lipid and the largest decrease in the interactions between lipids and lipids and between lipids and water, leading to the largest change in the liposome structure. We also studied how the degree of lipid tail unsaturation affects liposome structure changes with temperatures. Due to the C3 kinks in the unsaturated lipid tails, the distribution density of unsaturated lipids is not as high as saturate ones, leading to smaller attraction interactions and consequently larger liposome structure change with temperature. The obtained results are useful for the liposomal DDS design for the purpose of improving DDS performances and delivery outcomes.

## 1. Introduction

Liposomes, spherical bilayer lipid-containing vesicles [1,2], are promising nanocarriers for delivering drugs [3,4,5,6,7], owing to advantages such as reducing drug toxicity, increasing drug solubility, controlling delivery processes (e.g., targeting tumor sites, prolonging drug-release time and minimizing systemic exposure, etc.), and improving drug therapeutic efficacy [8,9,10,11,12,13].

When drug delivery systems (DDS) constructed with liposomes arrive at the targeted tumor cells, several methods can be applied to loosen or break the liposome structure and release drugs. One of the most commonly used methods is to heat the liposomal DDS by letting the gold nanoparticles or other light-absorbing substances, such as indocyanine green (ICG) [14,15,16,17,18,19,20,21], that partition in various portions (inner water core, lipid bilayer or the outside of the liposome) of the liposome absorb light irradiation. By designing wet lab experiments, researchers can regulate the distribution of light-absorbing material. Researchers wanted to learn which portion leads to the largest liposome structure change in response to the same temperature variation in order to assist the design of liposomal DDS. They did experimental research [16,22,23,24,25,26] and observed that heating the lipid bilayer and inner water core causes larger liposome structure change than heating the outside of liposome [16]. Nevertheless, even though light-absorbing substances in different portions are irradiated for the same time and with the same irradiation intensity, it is impossible to determine if the three portions have the same temperature variation. Wet lab experiments cannot help researchers obtain the related micromechanism and molecular-level details of the impacts of temperature changes on the liposome structure.

The saturation and unsaturation of acyl strands of lipids also have significant impacts on liposome structure change with temperature and therefore need in-depth molecular-level insights.

The molecular dynamics (MD) method is extensively employed by researchers to simulate the structures and behaviors of complex systems, in order to obtain related micromechanisms and in-depth molecular-level insights which are difficult or impossible to obtain through classical wet lab experiments [27]. In addition, MD simulations can precisely set and modify temperature, pressure, system compositions, etc., allowing for a systematic exploration of how variables such as temperature, lipid saturation and unsaturation may impact the liposome structure [28].

As a result, researchers use the MD method to simulate liposomal systems, but most researchers tend to simulate only a planar bilayer structure composed of lipids maybe due to the difficulties in establishing the structure of a whole spherical liposome as well as in simulating a system as large as a whole spherical liposome [29,30]. Amărandi et al. demonstrated that such simplification strategy may give wrong simulation results contrary to the experimental results [31]. Though Jämbeck et al. [32] and Zhu et al. [33] established a whole spherical liposome model with a diameter of about a dozen nanometers, they did not simulate the temperature-related properties of the liposome model. Therefore, currently there is a lack of research on simulating the structure change of a whole spherical liposome in response to temperature variations.

So, we used the MD simulation method to investigate which portion, the inner water core, the lipid bilayer or the outside, can lead to the largest liposome structure change under the same temperature variation. We established the whole spherical structure of a liposome composed of widely used 1,2-dipalmitoyl-sn-glycero-3-phosphocholine (DPPC), simulated how its structure changes with temperatures and obtained molecular-scale research results. We furthermore investigated how the saturation and unsaturation of lipid tails impact liposome structure change with temperature, comparing the differences in structure changes with the temperature of liposomes composed of saturate DPPC and unsaturated 1,2-dioleoyl-sn-glycero-3-phosphocholine (DOPC) and 1-palmitoyl-2-oleoyl-sn-glycero-3-phosphocholine (POPC). In sum, we investigated which portion’s temperature increases cause the largest structure changes and its mechanisms, and the impacts of the degree of saturation of lipid tails on liposome structure change with temperature and the relevant mechanisms.

## 2. Simulation Methods

Using the coarse graining method that Marrink et al. [34,35,36,37] proposed, we obtained the coarse-grained structural formulas of DPPC, DOPC and POPC, as shown in Figure S1 (Supplementary Materials). The coarse-grained MARTINI 2.1 force field was used to describe the three lipids and MARTINI water (4 molecules per bead).

It is more difficult to construct the model structure of a whole spherical liposome composed of solely DPPC, DOPC or POPC than that of a planar lipid bilayer. We employed the GROMACS simulation package to perform the simulations. We wrote code ourselves (Supplementary Materials) and established successfully the whole spherical structure of a liposome with a diameter of 30 nm. Liposomes with a size of 30 nm in diameter are apparently larger than those Jämbeck et al. and Zhu et al. established [32,33]. Furthermore, the micromechanisms derived are irrelevant with the size of liposomes, so it can be applied to liposomes with a larger size. Given the value of the areas per lipid (APL) of ~0.46 nm^2^ [38], this results in 5128 lipids in the outer leaflet of the lipid bilayer and 3638 lipids in the inner leaflet. Our research focuses on the temperature impacts on liposome structure changes, so simple liposomal systems with a single composition can help us find the correct micromechanisms behind, which are irrelevant with the number of compositions and of course can be applied to complex liposomal systems composed of multiple compositions. According to the volume of water beads, the inner water core has 67,522 water beads and the outside of the liposome contains 334,358 ones. The initial side length of the cubic simulation box was set as 40 nm. Energy minimization runs were performed with steepest descent method to remove improper configurations like the overlaps of bead positions. Leap-frog (LF) algorithm was used. The electrostatic and Lennard-Jones potentials are computed with the Verlet cutoff scheme, with a cutoff length of 1.2 nm for both potentials. Then a dynamic equilibrium simulation was carried out for 300 ps in an isothermal–isobaric (NPT) ensemble by using 10 fs time steps, with the pressure and temperature set as 1 bar and 300 K, respectively. The v-rescale thermostat and Parrinello−Rahman barostat were used for both the dynamic equilibrium and production runs. The v-rescale thermostat is similar to the Berendsen one, but the former can ensure the creation of the correct NPT systems. The parameter “tc-grps” was used to set the three portions (components), the inner water core, the lipid bilayer, and the outside (water), as separate groups for temperature coupling. Separate thermostat groups were used. The parameter “ref_t” was used to set the temperature of each group. The temperature coupling corresponds to various components of the systems, not to regions. The coupling constant, “tou_p”, was set the value of 13. Semi-isotropic pressure coupling was applied. The final frame of the dynamic equilibrium simulation was used as the starting configurations for subsequent simulations to study liposome structure changes with temperatures. Then, we performed production simulations to explore the liposome structure change with temperature increased from 300 K to 390 K. From a molecular scale, a temperature below or above the transition temperature (Tm) is irrelevant to the simulation process [38], as the MARTINI 2.1 force field can be applied in the whole temperature range. After the temperature increase, the structure gradually changes from an initial gel/liquid state to a final liquid state, not an abrupt switch, so the simulations beginning at temperatures lower than the Tm of DPPC do not have an effect on the observed changes in the DPPC bilayer. The v-rescale thermostat was used to increase the temperature to 390 K directly. The 390 K simulations represent an accelerated condition intended to reveal deformation mechanisms rather than a realistic physiological temperature. Apparent notches in bilayer may appear in a several-hundred-nanosecond run after the temperature increases from 300 K to 390 K. Simulations with temperature increased to 330 K were performed as well, and similar results were obtained, but such runs were rather computationally expensive and unnecessary (several-microsecond runs are needed to observe a notch in the membranes of liposomes). As a result, it is unnecessary for the temperature to be increased in a stepwise manner to a lower temperature first.

The purpose of the study is to determine the same temperature increase in which portion leads to the largest liposome structure change, focusing on the liposome properties itself; therefore, including light-absorbing substances is unnecessary and doing so cannot ensure that the corresponding portion gets the same increase in temperature.

When the temperature of the portions is 330 K, the temperature gradient is lower than in the case of 390 K, but similar results are obtained. The computed APL of lipid membranes of DPPC, POPC and DOPC liposomes heated to 330 K in the lipid bilayer are 0.64, 0.69 and 0.79 nm^2^, respectively, in ascending order and in agreement with the order of the corresponding liposomes heated to 390 K in the lipid bilayer. When light-absorbing substances that partition in various portions of the liposome absorb light irradiation, they are heated and their moving speeds are increased. Such increased speeds are transferred to nearby particles of that portion by collisions with them, leading to temperature increase in the corresponding portion when most of the particles of the portion get higher speed. It is true that when light-absorbing substances in the lipid bilayer heat that portion, the water nearby can be affected; therefore, we analyzed the effects by calculating the radius distribution function of water around lipids. Nevertheless, the majority of the water particles in inner water core and outside do not have chances to collide with light-absorbing substances or lipid particles with higher temperatures, so the temperature of that portion stays unchanged.

## 3. Results and Discussion

The snapshots of the final frame (300 ps) of the dynamic equilibrium simulation of the DPPC liposome system at 300 K are presented in Figure S2. The frame was used for subsequent MD simulations to study liposome structure changes with temperature. The frames of the dynamic equilibrium simulation of liposomal systems corresponding to the run times of 150 ps and 450 ps were also used for subsequent MD simulations to study liposome structure changes with temperatures, and similar results were achieved. The APL of the lipid membranes of the frames corresponding to the run times of 150 ps, 300 ps and 450 ps are 0.47, 0.48 and 0.48 nm^2^, respectively; therefore, the liposome structure has been stabilized.

**Figure 1:**
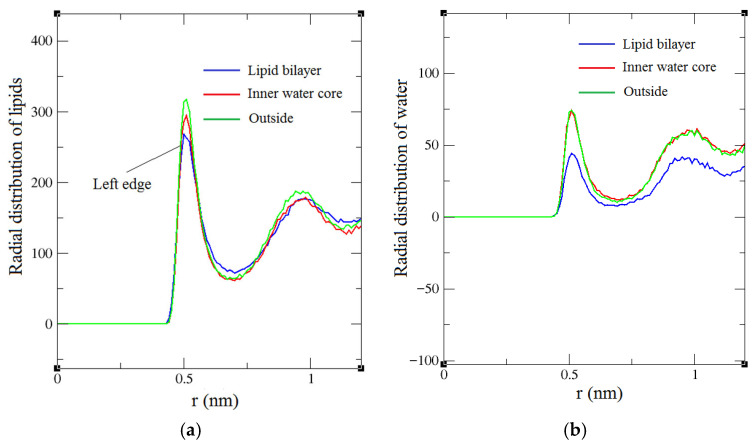

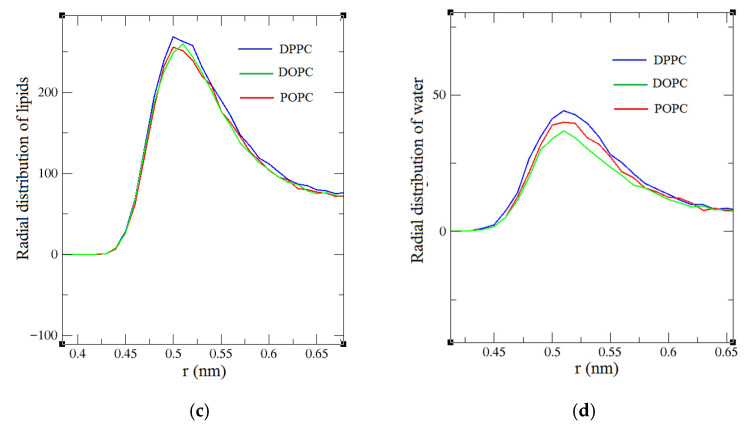
The radius distribution of (**a**) lipids and (**b**) water around a lipid after the temperature of the three portions of the DPPC liposome are increased from 300 K to 390 K, and 230 ns of production runs are performed. The radius distribution of (**c**) lipids and (**d**) water around a lipid after the temperature of the lipid bilayer of DPPC, DOPC and POPC liposomes are increased from 300 K to 390 K, and 230 ns of production runs are performed. r represents the distance from a reference lipid, and the radial distribution value represents the number of lipids or water whose distance from the reference lipid is in the range of [r − 0.005 nm, r + 0.005 nm].

**Figure 2:**
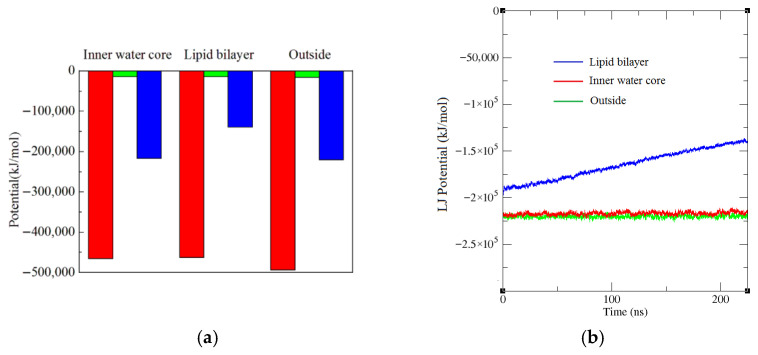
(**a**) The Lennard-Jones potential (red column) and electrostatic potential (green column) between lipids and lipids and the Lennard-Jones potential (blue column) between lipids and water, after the temperature of its three portions are increased from 300 K to 390 K, and 230 ns of production runs are performed. The electrostatic potential between lipids and water is zero, so it is not presented. (**b**) The Lennard-Jones potential between lipids and water, after the temperature of the three portions are increased from 300 K to 390 K, as a function of production run time. The data were extracted from the “ener.edr” file created in the process of production simulations using the “gmx energy” command. The electrostatic and Lennard-Jones potentials are computed with the Verlet cutoff scheme. The reported values represent averages over the full trajectory.

**Figure 3:**
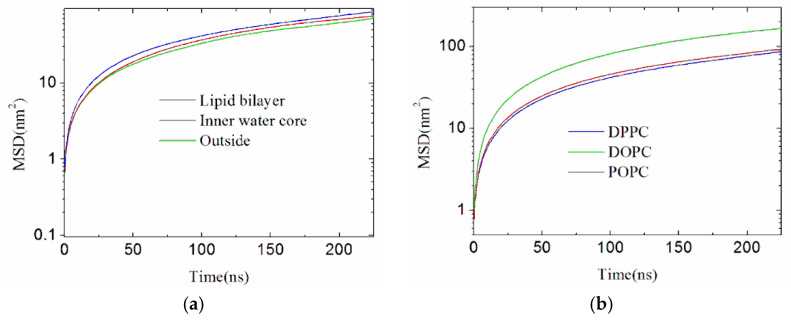
(**a**) The fluidity of the lipids after the temperature of the three portions is increased from 300 K to 390 K as a function of production run time. The non-equilibrium lateral diffusion coefficients of lipids are 0.0597, 0.0563 and 0.0496 (10^−5^ cm^2^/s) for temperature increases in the lipid bilayer, the inner water core and the outside, respectively. (**b**) The fluidity of the lipids after the temperature of the lipid bilayer of DPPC, DOPC and POPC liposomes are increased from 300 K to 390 K, as a function of production run time. The non-equilibrium lateral diffusion coefficients of lipids are 0.0597, 0.1213 and 0.0650 (10^−5^ cm^2^/s) for DPPC, DOPC and POPC, respectively.

MD study was performed to simulate how the structure of the DPPC liposome changes after the temperature of its three portions, the inner water core, the lipid bilayer and the outside, are increased from 300 K to 390 K, respectively. Figure S3 shows the snapshots of the simulated liposome systems corresponding to the three cases after 230 ns of production runs, which corresponds to the full production trajectory. It is observed that the temperature increase in the lipid bilayer introduces the largest changes in the liposome structure. The liposome apparently has the largest change in its appearance and there is even a notch whose size is around 2.5 nm in diameter. We calculated the radius distribution of lipids and water around a lipid in all the three simulated cases and presented the results in Figure 1. The radial distance is the minimum distance between any beads of the molecules. Only the comparison of the area of the first crest and the position of its left edge is necessary, because the magnitude of the interaction among beads decreases abruptly with increasing distances and therefore the contribution of other crests is negligible. The first crest determines the magnitude of the lipid packing density. The most important thing is to compare the position of the left edge of the first crest. At a low distance near zero, the left edges of various crests usually merge with one another. With distance increasing, the left edges will then separate and the one at the left is at a closer distance than the others (Figure 1). Usually, if the left edge of a crest is at a closer distance than another one, the crest will have a larger area as well. People also use APL directly to compare the lipid packing density. According to Figure 1, Figure 2 and Figure 3, compared to the other two cases, the temperature increase in the lipid bilayer leads to the lowest distribution densities of lipids and water around a lipid and the bilayer membrane is consequently the sparsest; the attraction interaction between lipids and lipids and that between lipids and water is the smallest (Figure 2, corresponding to largest interaction potential), and the fluidity of the lipid membrane is the largest (Figure 3). The most important factor that impacts liposome structure stability is the attraction interactions between lipids and lipids and between water and lipids, whose magnitude is determined by the lipid and water distribution density around a lipid. From Figure 1, the temperature change in the inner water core brings about sparser lipid membrane than that in the outside of a liposome, because the corresponding first crest area is smaller and its left edge is at a further distance; the distribution density of water around a lipid is also lower than in the case of the temperature increase in the outside, and the interaction between lipids and lipids and between lipids and water is lower as well. In sum, the temperature change in the inner water core introduces larger liposome structure changes than that in the outside of the liposome. The computed APL of lipid membranes of liposomes heated in the lipid bilayer, inner water core and outside are 0.73, 0.56 and 0.52 nm^2^, respectively, in descending order and in agreement with the order of lipid packing density derived from Figure 1.

Figure 3 shows the mean square displacement (MSD) of lipids in all the three simulated cases, which is a lipid mobility measure of DPPC membranes. The temperature increase in the lipid bilayer leads to the highest lipid fluidity, followed by that in the inner water core, with that in the outside of the liposome being the lowest.

Figure 2b shows that compared to the other two cases, after the temperature increase from 300 K to 390 K in the lipid bilayer, the attraction interaction between lipids and water decreases apparently and continuously, furthermore indicating that it introduces the largest impacts.

The analysis above suggests the micromechanism of the liposome structure changes due to temperature change in all the three cases. Compared to the other two cases, the temperature increase in the lipid bilayer leads to the largest increase in strand sway amplitude [39]. According to Amărandi et al. [31], the inclusion of cholesterol as a composition of the liposome will stabilize the liposome and improve lipid packing density, so it will decrease the changes in strand sway amplitude but will not reverse the change trend. Attributed to the highest lipid fluidity according to Figure 3, the temperature increase in the bilayer causes the largest changes in bilayer lipid positions, the largest decrease in the distribution density of lipids and water around a lipid, the largest decrease in the interactions between lipids and lipids and between lipids and water, leading to the largest changes in the liposome structure.

We studied the differences in the structure changes in DPPC, DOPC and POPC liposomes with temperatures increased from 300 K to 390 K, in order to study the effects of the degree of unsaturation of lipid tails on liposome structure change with temperature.

**Figure 4:**
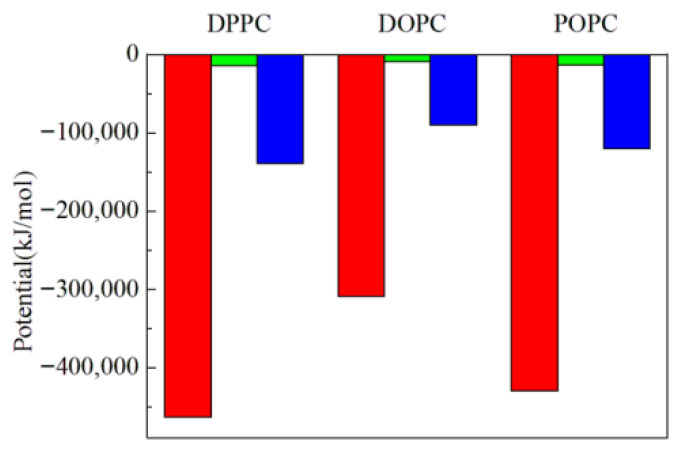
The Lennard-Jones potential (red column) and electrostatic potential (green column) between lipids and lipids and the Lennard-Jones potential (blue column) between lipids and water, after the temperature of the lipid bilayer of DPPC, DOPC and POPC liposomes is increased from 300 K to 390 K, and 230 ns of production runs are performed.

As presented in Figures S3 and S4, in all the three cases, the liposomes have various changes in their appearances and even observable notches exit after 230 ns of production runs. The DOPC liposome has the largest notches due to the same temperature change in the lipid bilayer, followed by POPC, with DPPC having the smallest. Figure S5 illustrates the formation process of the large notches of the DOPC liposome. The calculated results of the radius distribution of lipids and water around a lipid are presented in Figure 1c,d. From Figure 1, the area of the crest corresponding to the POPC liposome is similar to the DOPC liposome, but the left edge of the crest corresponding to the DOPC liposome is at a further distance, so the distribution density of lipids around a lipid of the DOPC liposome is smaller than the POPC liposome. It is observed that the DOPC liposome has the lowest distribution densities of lipids and water around a lipid (the sparsest bilayer membrane), and the lowest attraction interactions between lipids and lipids and that between lipids and water according to Figure 4 (corresponding to the largest interaction potentials), followed by the POPC liposome, with the DPPC liposome shows the highest values. The computed APL of lipid membranes of DPPC, POPC and DOPC liposomes heated in the lipid bilayer are 0.73, 0.80 and 0.91 nm^2^, respectively, in ascending order and in agreement with the order of lipid packing density derived from Figure 1. Figure 3b shows that the MSD (fluidity) of the DOPC lipids of the DOPC liposome is the largest, followed by POPC, with DPPC the smallest.

Due to the stiffness of unsaturated lipid tails, the C3 kinks in the tails are therefore induced [40] (Figure S6). As long as the lipid tail contains C3 beads with unsaturated bonds, the corresponding angle has a low average value of around 122°. This suggests the underlying micromechanism. According to radius distribution data and APL values, due to the C3 kinks, the tails of unsaturated lipids cannot keep too close to one another and the distribution density of unsaturated lipids is not as high as saturate ones, leading to smaller attraction interactions, and therefore larger liposome structure changes with temperature. Furthermore, such effects increase with the degree of lipid tail unsaturation according to the comparison between the DOPC and POPC liposomes. As a result, to design a thermo-responsive liposome with a good drug-release property upon light irradiation, unsaturated lipids with a proper degree of tail unsaturation should be used to prepare liposomes, or unsaturated and saturated lipids should be used together to obtain a liposome whose stability and thermo-responsiveness both meet requirements. People tend to think that due to the existence of C3 kinks, the two end beads of the same lipid molecule should have a larger distance. We calculated the average distance between the end beads 8 and 12 (Figure S1) of the same lipid molecule in DPPC, DOPC and POPC, and the results are 1.51 nm, 1.43 nm and 1.50 nm, respectively. As a result, the existence of C3 kinks does not necessarily cause a larger distance between the two end beads of the same lipid molecule.

## 4. Conclusions

Liposomes are promising vehicles to be used for delivering drugs. Currently there is a lack of research on simulating the structure change of a whole spherical liposome due to temperature variations. Therefore, we established the whole spherical structure of the liposome, simulated its change with temperatures and obtained molecular-level research results. We got to learn that compared to the temperature increase in other portions of a liposome, that increase in the lipid bilayer leads to the largest increase in lipid strand sway amplitude, the largest changes in lipid positions, the largest decrease in the distribution density of lipids and water around a lipid and the largest decrease in the interactions between lipids and lipids and between lipids and water, inducing the largest change in the liposome structure. We also studied how the degree of lipid tail unsaturation affects liposome structure change with temperature. Due to the stiffness of unsaturated lipid tails, the C3 kinks in the tails are induced (C3 kinks do not necessarily lead to a larger distance between the two end beads of the same lipid molecule, though people tend to think it is so). Consequently, the tails of unsaturated lipids cannot keep too close to one another and the distribution density of unsaturated lipids is not as high as saturate ones, leading to smaller attraction interactions and larger liposome structure change with temperature.

The obtained results above are useful for the liposomal DDS design for improved DDS performance and delivery outcomes. To design a liposome with a good drug-release property upon light irradiation, the irradiation-absorbing substances should partition in the lipid bilayer portion. Furthermore, people should use unsaturated lipids with proper degree of tail unsaturation to prepare liposomes or use unsaturated and saturated lipids together in a certain ratio to obtain a liposome whose stability and thermo-responsiveness both meet requirements.

The obtained micromechanisms and molecule-scale insights provide a profound understanding of the liposomal systems and offer guidance for future research. In the future, researchers can explore the impacts of other factors, such as pH values, on liposome structure changes and obtain the relevant mechanisms, consolidating the results of this study.

## Data Availability

The raw/processed data required to reproduce these findings will be provided upon the requests.

## Supplementary Materials

The following supporting information can be downloaded at: https://www.mdpi.com/article/10.3390/membranes16040124/s1, Figure S1: The coarse-grained structural formulas of (a) DPPC, (b) DOPC and (c) POPC. “Q0” and “Qa” represent charged coarse grains (beads), with “0” and “a” indicating that the beads have neither acceptors nor donors for hydrogen bonds, and only acceptors, respectively. “Na” corresponds to nonpolar beads that contain only acceptors for hydrogen bonds. C1 and C3 represent hydrophobic beads, with C1 containing no unsaturated bonds, but on the contrary C3 containing unsaturated bonds. It is observed from Figure S1 that one DOPC molecule has two lipid tails containing unsaturated bonds, while one POPC molecule only has one such tail. Figure S2: Snapshot of the simulated DPPC liposome at 300 K. Figure S3: The snapshots of the simulated liposome systems after the temperature of its three portions, (a) inner water core, (b) lipid bilayer (the white circle marks the notch) and (c) outside, are improved from 300 K to 390 K, respectively, and 230 ns of production runs are performed. Note: for better clarity, only the inner leaflet is shown but the outer one and water are not presented. Figure S4: The snapshots of the simulated (a) DOPC (with notch in a diameter of around 10 nm) and (b) POPC (with notches in a diameter of around 4 nm) liposome systems after the temperature of the lipid bilayer are increased from 300 K to 390 K, respectively, and 230 ns of production runs are performed. Note: for better clarity, only the inner leaflet is shown, but the outer one and water are not presented. Figure S5: The snapshots of the simulated DOPC liposome systems after the temperature of the lipid bilayer are increased from 300 K to 390 K, and (a) 115 ns (b) 180 ns and 200 ns of production runs are performed, respectively. Note: for better clarity, only the inner leaflet is shown, but the outer one and water are not presented. Figure S6: The ratio distribution of (a) ∠5·6·7 and (b) ∠9·10·11 (Figure S1) after the temperature of the lipid bilayer of DPPC, DOPC and POPC liposomes are improved from 300 K to 390 K, and 230 ns of production runs are performed.
