# Supplementary material for: A Study of Liposome Structure Changes with Temperature Using Non-Equilibrium Molecular Dynamics Simulations
Source: Membranes (Basel). 2026 Mar 31;16(4):124. doi: 10.3390/membranes16040124 (PMC13117722; doi:10.3390/membranes16040124)
Supplement: Supplementary file 1 [file membranes-16-00124-s001.zip › Supplementary Materials.pdf]

Supplementary Materials for:

A study of liposome structure changes with temperature using non-equilibrium molecular dynamics simulations

G. Q. Yang*[a], Weibin Cai[b], Ying Wan[a]

[a]*College of Bioscience and Bioengineering, Jiangxi Agricultural University, Nanchang, Jiangxi, 330045, P. R. China.*

[b]*School of Chemical and Environmental Engineering, China University of Mining and Technology, Beijing 100083, P. R. China.*

*Corresponding author. Email: yq.1999@tsinghua.org.cn

Contents:

S1. The method to construct the liposome structure

S2. Figures

S3. The movie file showing the simulation trajectory of the DPPC liposome at 330K. (Similar results were obtained as 390K)

S1. The method to construct the liposome structure

This GROMACS software cannot construct the structure of a whole spherical liposome directly. We also used the CHARMM-GUI tool, but the constructed liposome structure has large holes in the bilayer membrane. Nevertheless our simulations need to start from an intact spherical liposome. Martini community developed the ts2cg tools, but the ts2cg tool-created vesicle has an apparent large gap between the two leaflets and lipids are not distributed evenly, deviating from the real vesicle and decreasing the robustness of the tool. As a result, we had to write code ourselves and established successfully the whole spherical structure of a liposome with a diameter of 30 nm. We constructed the liposome structure with the following algorithm. One lipid is placed along the x axis and the coordinates of the other ones are obtained by rotating it a certain angle around the z axis and another certain angle in the xy plane, so via coordinate transformation all the coordinates of other lipids of the vesicle can be obtained. After one leaflet is constructed, the other one can be constructed with the same method. This algorithm can ensure the evenness of vesicle membrane and the small gap between the two leaflets.

S2. Figures

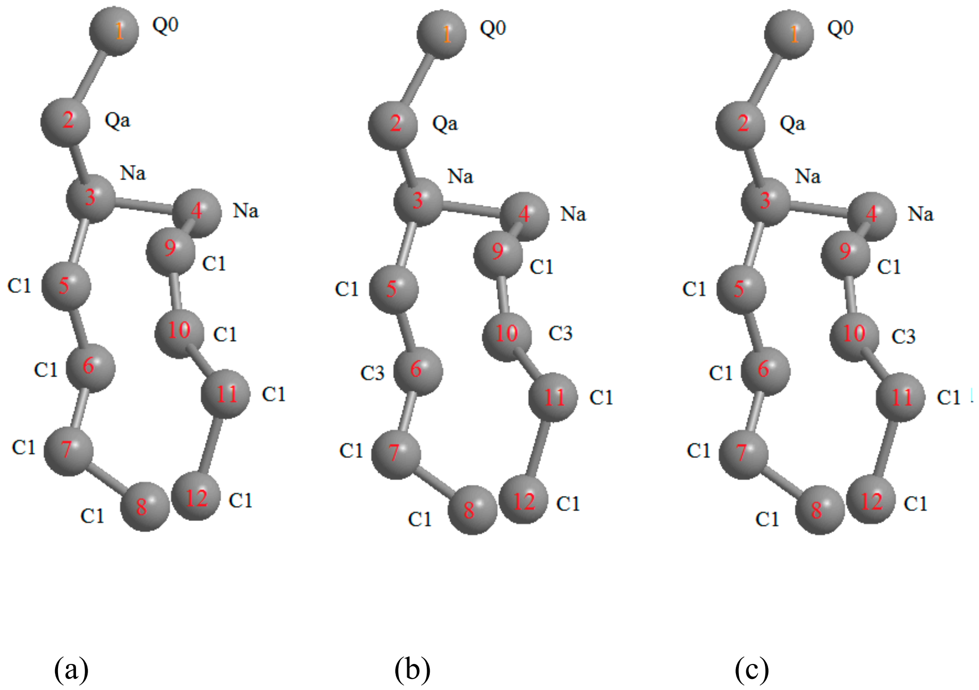

Figure S1. The coarse-grained structural formulas of (a)DPPC, (b)DOPC and (c)POPC.

"Q0" and "Qa" represent charged coarse grains (beads), with "0" and "a" indicating that the beads have neither acceptors nor donors for hydrogen bonds, and only acceptors, respectively. "Na" corresponds to nonpolar beads that contain only acceptors for hydrogen bonds. C1 and C3 represent hydrophobic beads, with C1 containing no unsaturated bonds, but on the contrary C3 containing unsaturated bonds. It is observed from Fig. S1 that one DOPC molecule has two lipid tails containing unsaturated bonds, while one POPC molecule only has one such tail.

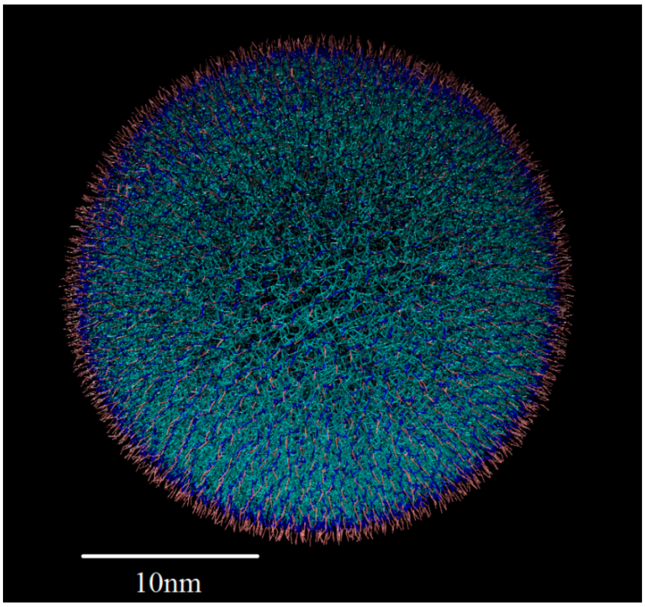

Figure S2. Snapshot of the simulated DPPC liposome at 300K.

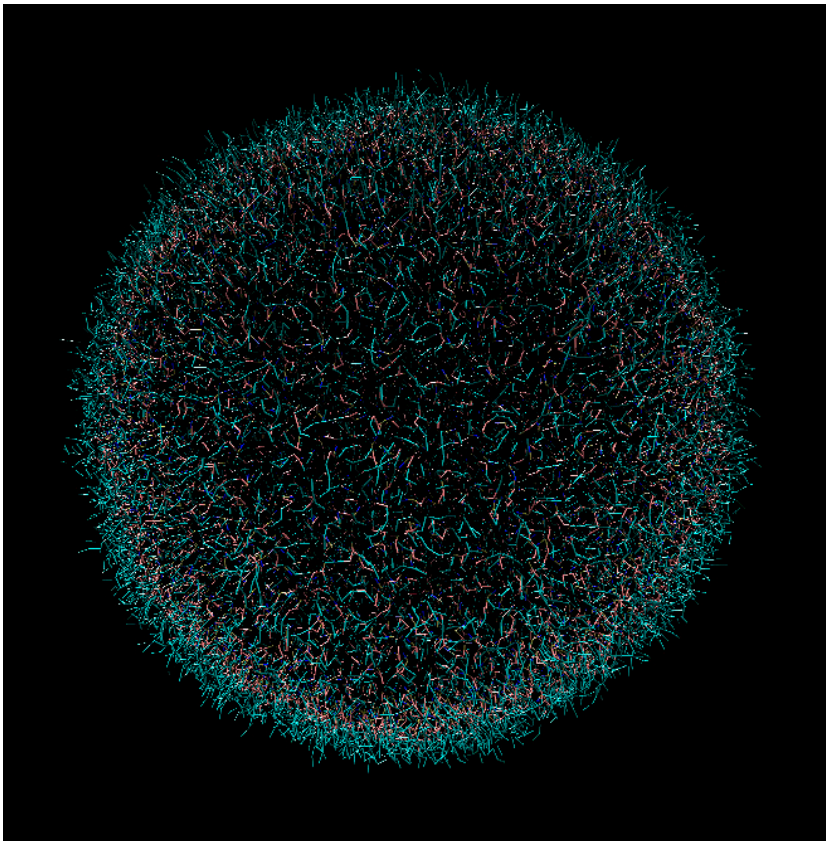

(a)

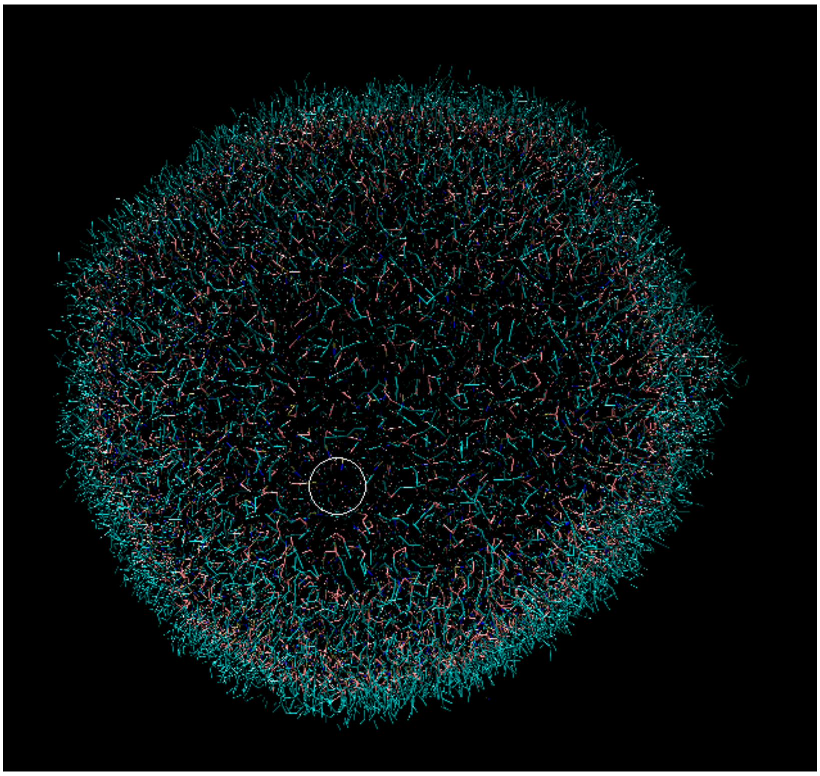

(b)

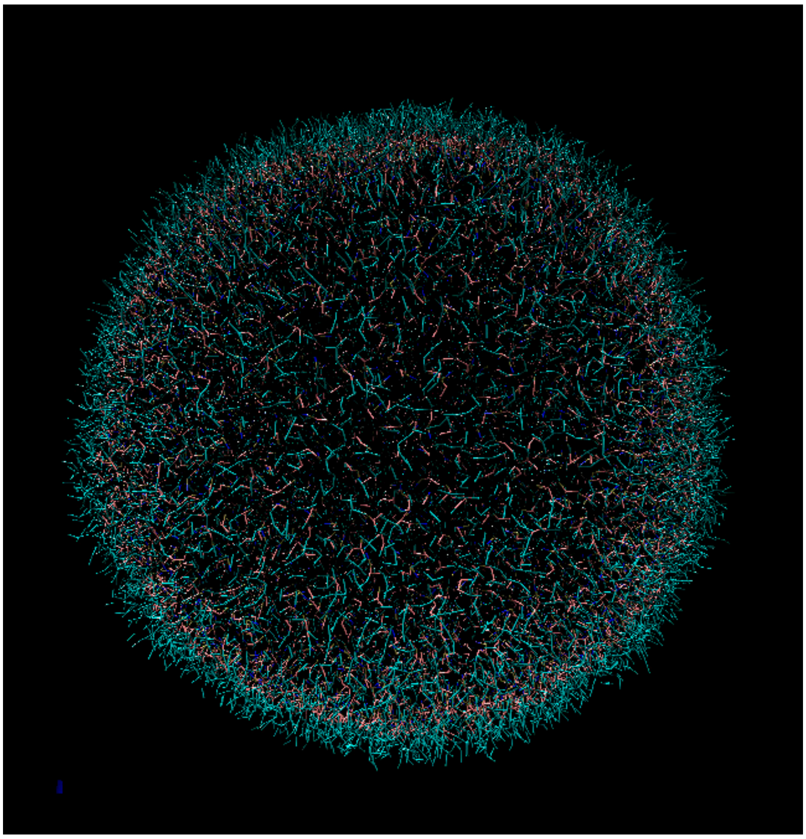

(c)

Figure S3. The snapshots of the simulated liposome systems after the temperature of its three portions, (a)inner water core, (b)lipid bilayer (the white circle marks the notch) and (c)outside, are improved from 300K to 390K, respectively, and 230ns of production runs are performed. Note: for better clarity, only the inner leaflet is shown but the outer one and water are not presented.

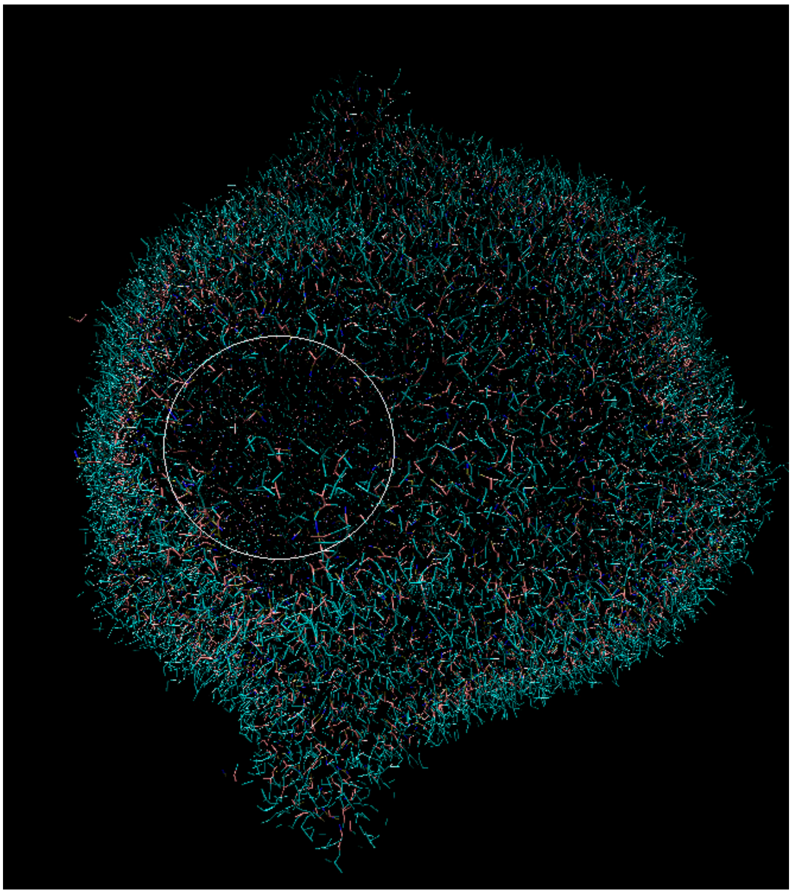

(a)

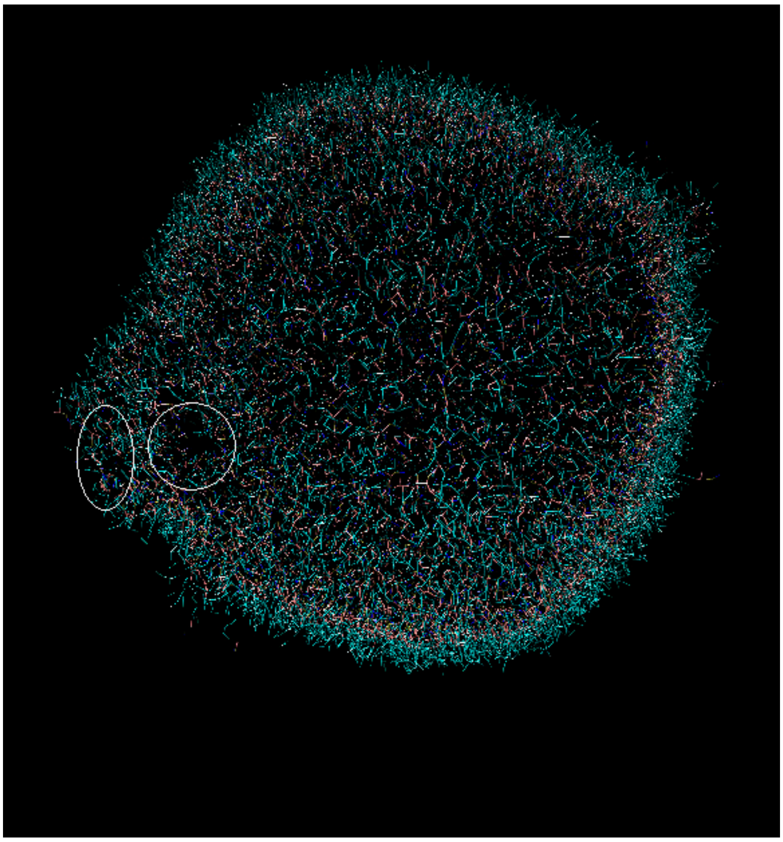

(b)

Figure S4. The snapshots of the simulated (a)DOPC (with notch in a diameter of around 10nm) and (b)POPC (with notches in a diameter of around 4nm) liposome systems after the temperature of the lipid bilayer are increased from 300K to 390K, respectively, and 230ns of production runs are performed. Note: for better clarity, only the inner leaflet is shown, but the outer one and water are not presented.

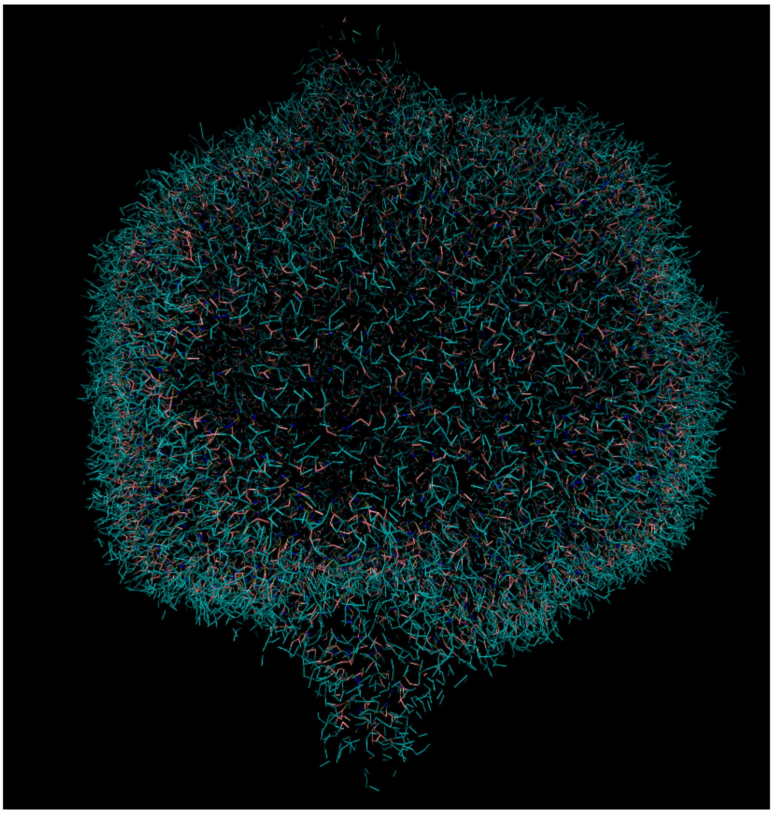

(a)

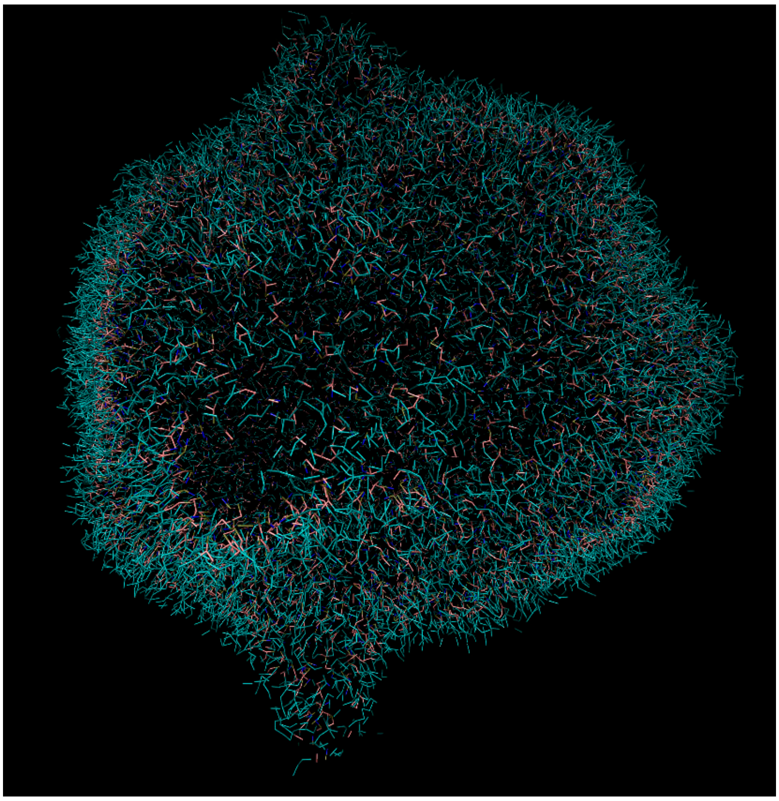

(b)

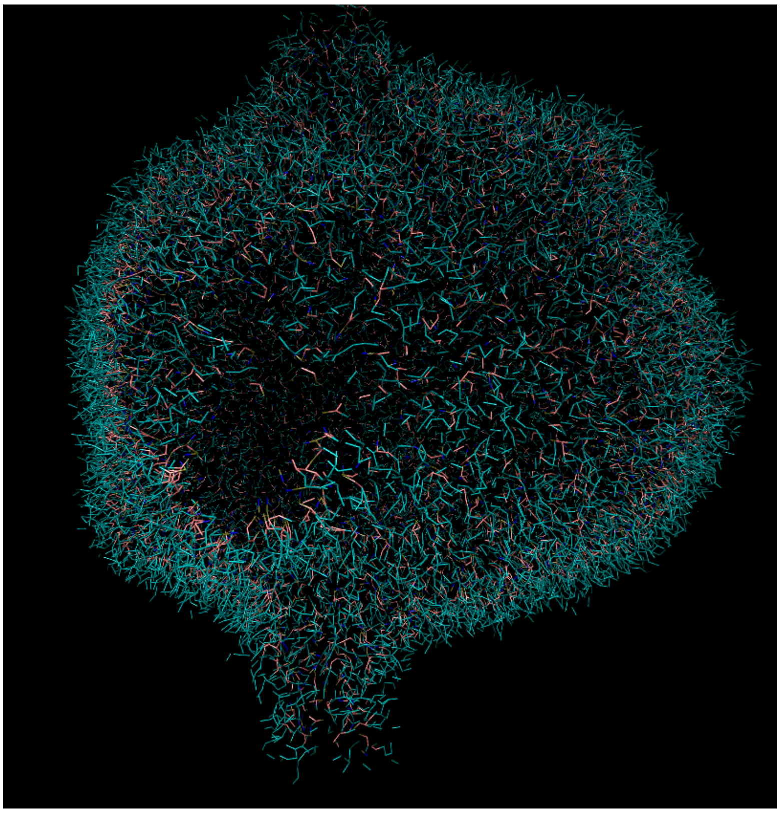

(c)

Figure S5. The snapshots of the simulated DOPC liposome systems after the temperature of the lipid bilayer are increased from 300K to 390K, and (a) 115ns (b) 180ns and 200ns of production runs are performed, respectively. Note: for better clarity, only the inner leaflet is shown, but the outer one and water are not presented.

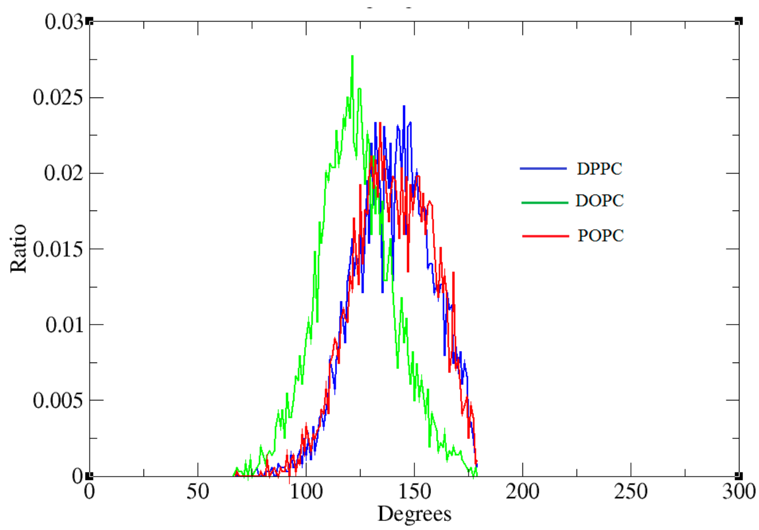

(a)

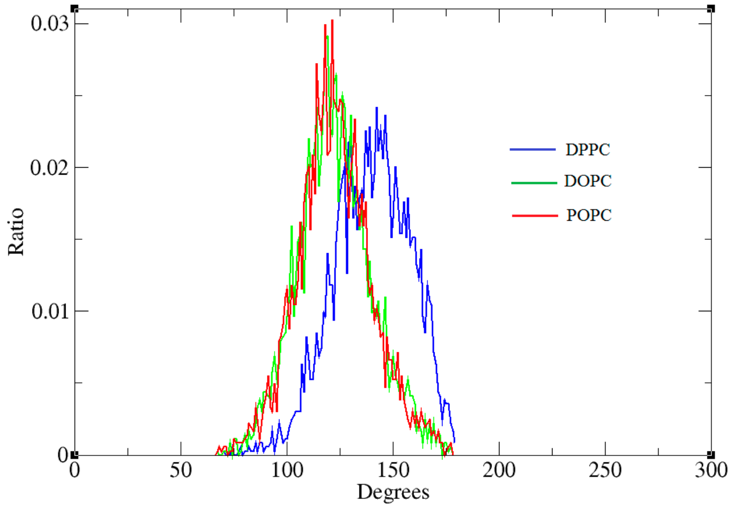

(b)

Figure S6. The ratio distribution of (a)∠5·6 7 and (b)∠9·10·11 (Fig. S1) after the temperature of the lipid bilayer of DPPC, DOPC and POPC liposomes are improved from 300K to 390K, and 230ns of production runs are performed. .
